# The role of whole network analysis in understanding health behaviours: a systematic review of smoking, alcohol use, physical activity, and diet

**DOI:** 10.3389/fpubh.2026.1834356

**Published:** 2026-06-18

**Authors:** Simone Sarti, Marco Terraneo, Roberta Gallina, David Consolazio

**Affiliations:** 1 Department of Social and Political Sciences, University of Milan, Milan, Italy; 2 Department of Sociology and Social Research, University of Study of Milano-Bicocca, Milan, Italy

**Keywords:** alcohol, diet, diffusion, health behaviours, peer influence, physical activity, smoking, social networks

## Abstract

**Background:**

Health behaviours such as smoking, alcohol use, physical activity, and dietary habits are not only individual choices but are influenced by social interactions and relational contexts. Recently, Whole Network Analysis (WNA) has become a powerful methodological tool to explore how these behaviours spread and become established within social systems. Unlike egocentric approaches, which focus on individuals’ personal networks, WNA captures the entire structure of social relations within a defined population.

**Methods:**

We conducted a systematic review of empirical studies applying WNA to four key health behaviours: smoking, alcohol consumption, physical activity, and diet. A thorough search was performed across six major academic databases for peer-reviewed articles published in English. We adhered to PRISMA 2020 guidelines when selecting and analysing the studies. Inclusion criteria included sociocentric data collection, a focus on behavioural processes, and empirical design.

**Results:**

A total of 27 studies met the inclusion criteria. Findings highlighted consistent structural mechanisms across behaviours, including peer influence, homophily, network cohesion, and centrality. WNA proved particularly effective in identifying behaviour clusters, co-evolutionary processes between networks and behaviours, and structural barriers or facilitators of behavioural diffusion. Longitudinal studies employing stochastic actor-oriented models provided especially valuable insights into disentangling selection and influence.

**Conclusion:**

WNA provides a distinctive perspective for understanding how health behaviours emerge and spread within social systems. This review underscores the importance of integrating WNA more systematically into public health research and intervention planning. By mapping entire social systems, WNA facilitates the identification of key actors, influential subgroups, and potential entry points for effective behavioural interventions. Future research should expand WNA applications to more diverse populations and settings and critically examine how social inequalities, such as socioeconomic status, gender, and contextual factors, interact with network dynamics to influence health behaviour trajectories.

## Introduction

Health behaviours such as smoking, alcohol use, physical activity, and dietary choices are critical determinants of individual and population-level health outcomes. In fact, they represent the main modifiable risk factors for non-communicable chronic diseases (NCDs), including cardiovascular diseases, cancer, chronic respiratory diseases, and type 2 diabetes, which are among the leading causes of morbidity and mortality globally (1). They are becoming an increasingly critical public health concern due to their high morbidity and mortality rates (2). Health-related behaviours are shaped by organisational, interpersonal, and individual factors, such as workplace environments, personal relationships, and material conditions. Furthermore, these behaviours are influenced by sociodemographic characteristics, including social class, gender, age, and ethnicity, which all affect daily experiences and opportunities for health-related choices (3).

Social networks, have emerged as a critical factor in shaping health behaviours, acting as channels through which information, influence, norms, and support circulate. Network characteristics, such as size, density, composition, and tie strength (for a detailed description of these measures, which describe how a network of relationships among individuals or groups is organized and functions, see Valente (4)), determine the degree to which individuals are exposed to behaviours, supported in changing existing habits, or constrained by prevailing norms. Evidence suggests that some health behaviours may be more susceptible to network influence than others. Behaviours that are socially visible and commonly performed in group settings, such as smoking, alcohol consumption, and physical activity, tend to show stronger peer influence effects than more private or individually regulated behaviours such as dietary practices (5–7). For instance, larger and more diverse networks have been associated with greater engagement in health-promoting behaviours, such as physical activity (8), as well as a lower number of cigarettes smoked or alcohol consumed (9).

Tie strength also plays an important role in shaping health behaviours. While weak ties may offer access to ideas, strong ties, such as family members or close friends, have a significant influence on daily behaviours (10, 11). Close family and friends tend to reinforce behaviours through shared environments and mutual influence, while broader friendship networks contribute to the diffusion of both protective and risky behaviours (6, 12). Social contexts, including schools, workplaces, and communities, shape the structure and content of social ties, influencing behavioural choices through exposure to collective norms and shared practices (10, 13).

The transmission of health behaviours occurs through various processes within social networks. Social norms, for instance, dictate what is considered acceptable within a group and shape individual behavioural intentions and practices (14, 15). Social learning occurs when individuals adopt behaviours modelled by influential peers (10, 11). Social support, both emotional and instrumental, can buffer stress and reinforce health-promoting behaviours (16), while peer influence and social facilitation can diminish perceived barriers and increase the desirability of both healthy and harmful behaviours. The centrality of an individual within a network further modulates exposure to these mechanisms: individuals in central positions are more visible, influential, and susceptible to the broader trends of their social network (13).

Empirical studies have applied these theoretical insights to understand a wide range of health behaviours, such as contraception use (17–20), the spread of infectious diseases like HIV (21–26), or even the transmission of behaviours related to COVID-19. Studies on vaccination uptake (27, 28) have also demonstrated how social networks influence the adoption and persistence of health behaviours.

This review, considering the Whole Network Analysis (WNA) approach, focuses on four key health behaviours that are of significant public health concern: smoking, alcohol consumption, physical activity, and diet-related behaviours – all of which are strongly associated with non-communicable chronic diseases, including cardiovascular diseases, cancer, chronic respiratory diseases, and type 2 diabetes (29). A growing body of research highlights that behaviours like smoking (10, 16, 30–32), alcohol consumption (11, 33), physical activity (9, 34–36), and dietary habits, which are closely linked to obesity (13, 37–39), are influenced by interpersonal relationships and the broader structure of social networks (4, 6, 37).

For example, the likelihood of quitting smoking is higher when a spouse or close friend also quits (10). Similarly, alcohol use is influenced by network patterns, with binge drinking behaviours propagating through networks up to three degrees of separation (11). Peer group structure in adolescence significantly shapes smoking initiation (31, 32), and some studies confirm that selection and influence processes jointly shape alcohol use, gambling, and substance co-use (14, 33). Physical activity and diet are also influenced by network dynamics. Individuals who are embedded in networks with active peers are more likely to engage in physical activity (34, 35, 40–42). Additionally, obesity is known to spread through social networks, as individuals are more likely to gain weight if a friend, sibling, or spouse becomes obese (37). Intergenerational patterns have also been demonstrated, where weight-related behaviours cluster across family and peer networks (43, 44).

### Methodological caveats

To capture these network-level dynamics, scholars have used Social Network Analysis (SNA), employing both Egocentric and WNA. Compared with traditional statistical approaches, network analysis offers the advantage of explicitly modelling interdependence between individuals rather than assuming observational independence. This allows researchers to examine how behaviours emerge through relational processes such as diffusion, peer influence, clustering, and social reinforcement. While Egocentric methods focus on individual-level ties (45), and are useful for understanding peer influence and social support, they fall short in capturing emergent group-level phenomena such as clustering, diffusion, or network-wide homophily (46).

WNA, also known as Sociocentric Analysis, examines the entire structure of social ties within a bounded population (e.g., classrooms, workplaces, communities). This method is particularly effective in exploring how behaviours spread, how norms are reinforced, and how specific network structures (e.g., centralisation, density) either promote or inhibit behaviour change (4, 47, 48).

WNA allows researchers to analyse how structural configurations, such as centrality, clustering, density, and modularity, relate to health behaviour adoption, diffusion, and change. It enables the detection of peer influence mechanisms, homophily patterns (i.e., the tendency to associate with similar others), and the presence of cohesive subgroups that may support or resist behavioural shifts. These capacities make WNA particularly well-suited for understanding health behaviours as socially situated phenomena.

Numerous studies have demonstrated that health behaviours tend to cluster within social networks, and that individuals’ positions in these networks can predict both exposure to risk and responsiveness to intervention (4, 48). For instance, adolescents embedded in smoking or drinking peer groups are more likely to initiate those behaviours themselves (49), while individuals in exercise-oriented cliques often maintain higher levels of physical activity. In addition, WNA has been used to identify central actors for intervention design (50), examine clustering and influence in schools (51), and study how social capital shapes behaviour in low- and middle-income countries (52).

Moreover, WNA mitigates the risk of endogeneity inherent in data collected through egocentric network analysis, where information about *alters* often relies solely on the *ego*’s reports. For instance, an obese individual may overestimate the weight of her/his contacts in an effort to protect his or her self-esteem (4, 53). On the other hand, WNA faces important limitations, including the high cost and methodological challenges associated with data collection, the practical constraint of observing only relatively small and bounded communities, and the consequent limited generalizability of its findings. In addition, it is often affected by missing or inaccurate relational data and raises ethical and privacy concerns that can further restrict access to complete network information.

Despite its promise, WNA seems underutilized in the health behaviours literature, especially compared to egocentric approaches. Most public health research still treats individuals as isolated units of analysis, occasionally accounting for social factors through demographic variables or reported social support. This individual-centric paradigm overlooks the structural and dynamic properties of the networks in which behaviours are embedded. Moreover, while the growing interest in network analysis is evident, its application to health behaviours, especially in complex social systems, has not reached its full potential.

Furthermore, the methodological demands of WNA, including the need for complete relational data, may have limited its diffusion. Yet, the added value of WNA lies precisely in its ability to model behaviour within the architecture of social relations, offering both explanatory and predictive insights.

Another limitation of current research is the tendency to apply WNA to only one health behaviour at a time, without considering whether similar network mechanisms operate across different domains. Comparative insights remain rare, and there is little synthesis of how peer selection, influence, or structural cohesion might generalise across smoking, diet, physical activity, and alcohol use. Furthermore, many studies do not explicitly distinguish between relational mechanisms (e.g., influence vs. selection). However, despite these strengths, network analysis remains less frequently used in public health research because it requires complex relational data collection, clear boundary specification, advanced analytical expertise, and often longitudinal designs, all of which can be resource-intensive.

### Study aims and objectives

The aim of this review is to provide a systematic synthesis of empirical studies that use WNA to study smoking, alcohol use, physical activity, and diet. Specifically, we address the following questions:

- How has WNA been applied to study health behaviours?
- What types of network structures, metrics, and models are most commonly used?
- What relational mechanisms (e.g., influence, selection, homophily) are identified across behaviours?
- What are the methodological and substantive implications of using WNA in public health?

By answering these questions, we aim to clarify the unique contribution of WNA in public health research. We argue that WNA enables a deeper understanding of how health behaviours are produced and reproduced within social systems, and that its systematic use can enhance both theoretical development and intervention strategies. In doing so, this work contributes to bridging the gap between network theory and health practice and to expanding the toolkit available for health behaviour change.

## Methods

### Study design

This review was conducted following the PRISMA 2020 guidelines (54) and aimed to systematically synthesise empirical research that employed WNA to investigate four health behaviours: smoking, alcohol use, physical activity, and diet. The methodological design emphasised rigour, transparency, and replicability.

We chose to focus exclusively on studies employing WNA, defined as research that collects and analyses relational data from all or nearly all members of a bounded population. These designs differ from egocentric or personal network studies by analysing entire sociometric systems, making it possible to capture emergent phenomena such as clustering, centralisation, and structural cohesion (4, 45, 55). By focusing on WNA, this review sought to understand how relational structures shape and are shaped by social networks.

Smoking, alcohol use, physical activity, and diet were selected because they are the four major modifiable lifestyle factors with well-established causal links to chronic diseases, are consistently and reliably measurable across populations, and are strongly shaped by peer norms and social structures (1). These features make them particularly suitable for network-based research, as compared to other lifestyle factors whose measurement or health relevance is less standardised.

### Eligibility criteria

To ensure focus and methodological coherence, we established strict eligibility criteria based on five dimensions: study design, network methodology, behavioural focus, publication standards, and language.

We applied the following inclusion criteria:

- Study design: The study must apply WNA within a bounded population (e.g., a school class, neighbourhood, dormitory, or workplace), ensuring coverage of at least 40% of the population to allow meaningful structural analysis (the 40% threshold was adopted as a pragmatic criterion to ensure sufficient network completeness for meaningful structural interpretation while retaining studies conducted in naturalistic settings where full participation is often difficult to achieve).
- Network methodology: The analysis must include the application of relational metrics or structural models such as density, degree centrality, closeness, betweenness, modularity, or stochastic actor-oriented models (SAOMs).
- Behavioural focus: The study must analyse at least one of the following behaviours as a primary or secondary outcome: smoking, alcohol consumption, physical activity, or dietary behaviour (including food choice and eating habits).
- Publication standards: Only peer-reviewed articles reporting original empirical research were considered.
- Language: Articles had to be published in English.

We used the following exclusion criteria:

- Studies relying exclusively on egocentric designs or partial networks without sociometric reconstruction of full networks. Furthermore, studies were excluded if they focused on non-human populations Review articles.
- Analyses centred on disease transmission or contact networks without behavioural outcomes.
- Intervention studies that mention network concepts without applying formal network analysis tools.
- Conference abstracts, dissertations, editorials, and other forms of grey literature.

These criteria ensured the inclusion of studies that rigorously apply WNA to understand health behaviours as relational phenomena within naturalistic or institutionalised social contexts.

### Information sources and search strategy

The literature search was conducted on January 12, 2025, using six major academic databases: PubMed, PsycINFO, CINAHL, Web of Science, Scopus, and Sociological Abstracts. These databases were selected for their comprehensive indexing of health, behavioural, and social science research.

The search string was constructed by combining four main conceptual domains:

- Network type: terms specific to whole network analysis, such as “whole network,” “whole network analysis,” “complete network,” “sociocentric,” “socio-centric,” “sociometric,” “sociomap,” and “sociogram”;
- Network methodology: general terms related to network analysis including “social network analysis,” “SNA,” “network analysis,” “network approach,” “network method,” “graph theory,” “structural network analysis,” and structural or dynamic network descriptors such as “peer influence,” “peer selection,” “social contagion,” “cluster,” “centrality,” “density,” and “homophily”;
- Health behaviours: keywords representing the four health behaviours of interest:
  *Smoking and tobacco use*: “smoking,” “tobacco,” “nicotine,” “cigarette*,” “vaping,” “vape,” “nonsmoker*,” “smoke*”;
  *Alcohol consumption*: “alcohol,” “alcohol use,” “alcohol abuse,” “alcohol consumption,” “drinking behaviour*,” “heavy drinking,” “binge drinking,” “drinker*”;
  *Physical activity*: “physical activity,” “exercise,” “workout,” “exercising,” “sedentary,” “sedentariness,” “physical inactivity”;
  *Diet and nutrition*: “obesity,” “overweight,” “diet*,” “eating behaviour*,” “eating pattern*,” “overeating,” “weight loss,” “body mass index,” “unhealthy eating,” “healthy eating,” “fast food consumption”;
- Conceptual framing: to capture broader conceptualisations, we included terms such as “health behaviour*,” “behavioural change,” “risk-taking behaviour*,” “social behaviour*,” and “human behaviour*.”

All keywords were searched within the title and abstract fields and combined using Boolean operators (“AND”/"OR”) to identify studies at the intersection of social network methods, whole network approaches, and health behaviours. The complete search strings for each database are available from the authors upon request.

### Study selection process

All retrieved citations were imported into Mendeley for deduplication, then transferred into a shared screening platform. Two of the authors independently screened titles and abstracts based on the inclusion criteria. Any discrepancies were discussed and resolved with input from a third author when necessary. Additionally, a fourth author reviewed the entire procedure to ensure consistency and accuracy.

**Figure 1:**
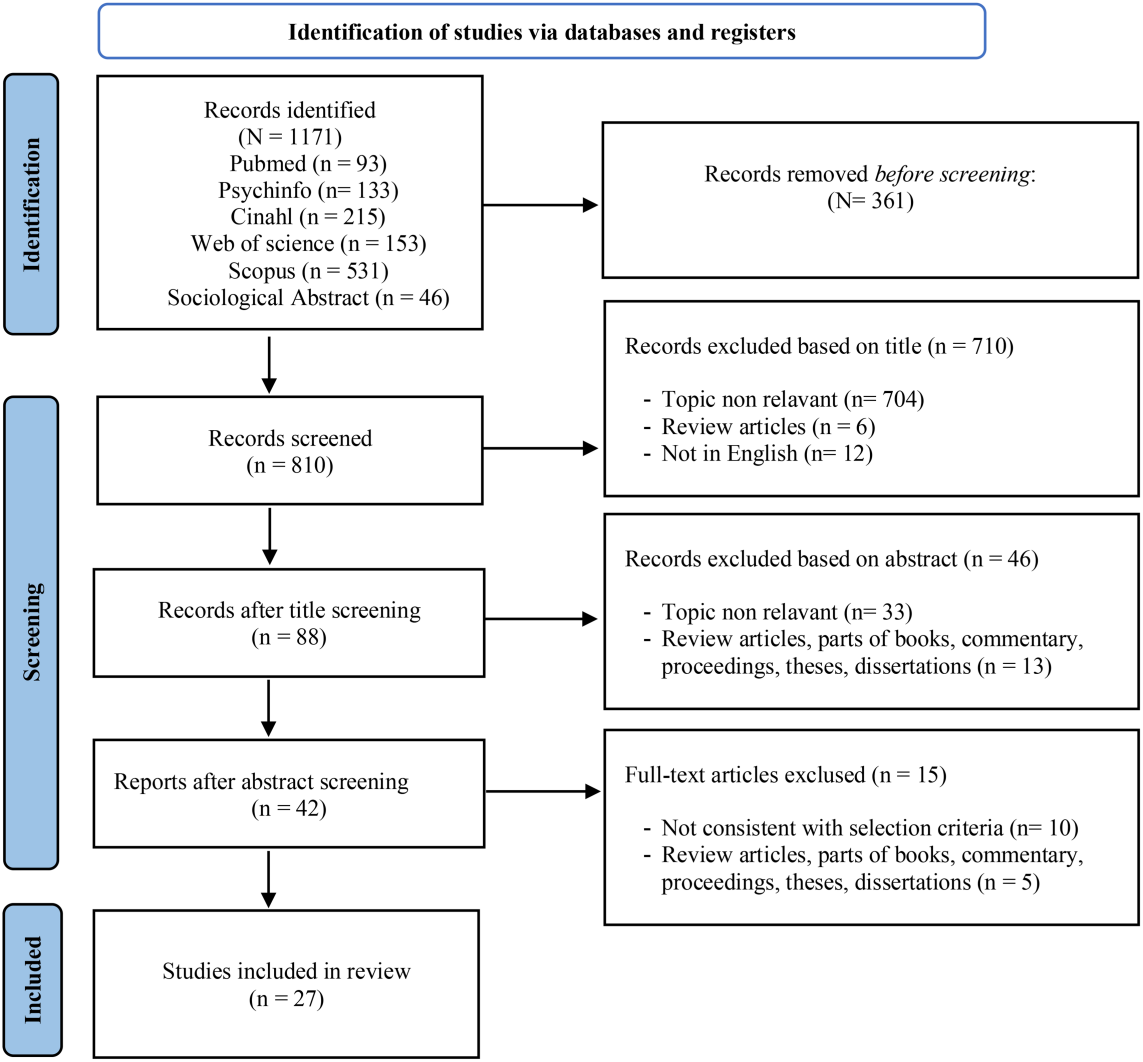
PRISMA flow diagram illustrating the study selection process.

Following title/abstract screening, full texts of potentially eligible studies were retrieved and assessed in detail. Reasons for exclusion were documented at the full-text level. The screening process is summarised in a PRISMA flow diagram (Figure 1).

Out of an initial pool of 1,171 records, 810 unique articles were screened by title and abstract. Of these, 42 full-text articles were assessed for eligibility, and 27 studies were included in the final synthesis.

### Data extraction and coding

A standardised data extraction form was developed and piloted. It captured:

- Study details: author(s), year of publication, and country of study
- Population characteristics: social context (e.g., school, workplace, community), and sample size and age group
- Health behaviour focus: primary behavioural outcome investigated (i.e., smoking, alcohol consumption, physical activity, diet)
- Network construction methods: tools and procedures used to elicit relational data (e.g., rosters, name generators, types of ties);
- Network metrics and analytical models used: structural indicators applied (e.g., degree, centrality, density, modularity, cohesion) and types of statistical or network modelling approaches used (e.g., regression analysis, stochastic actor-oriented models)
- Key findings: main behavioural outcomes and results of the network analysis
- Identified network mechanisms: social processes explored in relation to behaviours, including peer influence, selection and homophily, clustering/cohesion, and structural position (e.g., centrality or brokerage)
- Study limitations and methodological quality indicators: information on network boundary definition, response rate, handling of missing data, and longitudinal design

Reviewers extracted data, resolving inconsistencies through discussion. Studies were coded using thematic categories derived from prior research on network mechanisms in health.

### Risk of bias and quality assessment

While no universal risk-of-bias tool exists for WNA studies, we assessed methodological quality using the following criteria:

- Completeness of network data (e.g., participation rate ≥40%)
- Boundary clarity (well-defined and bounded populations)
- Network construction methods (e.g., validated name generators or fixed rosters)
- Appropriateness of metrics and modelling techniques
- Study design (e.g., longitudinal vs. cross-sectional)
- Behavioural measurement (e.g., validated scales, objective indicators)
- Sample size and nomination process (coverage and reliability of peer reports)

Each study was assigned a quality rating (Acceptable, Robust, or Strong) across these dimensions, and a cumulative score was calculated to derive an overall quality classification. Studies were not excluded based on quality, but methodological limitations were recorded and considered in the narrative synthesis. For instance, studies using cross-sectional designs were noted to have weaker causal inference capacity compared to longitudinal models.

### Synthesis approach

Given heterogeneity in designs and outcomes, a meta-analysis was not feasible. We employed a narrative synthesis, structured around recurring network social dynamics rather than behaviour type. This approach allowed us to compare findings across settings, populations, and behaviours.

Five central social processes guided our synthesis:

- Peer influence (behavioural convergence): behavioural change from exposure to connected peers.
- Selection and homophily: the tendency of individuals to form ties with others who share similar behaviours or characteristics.
- Cohesion and clustering: the formation of tightly connected subgroups that reinforce shared norms.
- Centrality and diffusion: the influence exerted by highly connected or strategically positioned individuals in spreading behaviours through the network.
- Brokerage and bridging roles: actors who connect otherwise separate subgroups and facilitate cross-group behavioural diffusion.

This thematic structure enabled identification of cross-behavioural patterns, emerging network configurations, and methodological gaps (4).

## Results

The final sample consisted of 27 empirical studies that applied WNA to examine one or more health behaviours: smoking, alcohol consumption, physical activity, and diet.

Findings are presented in Table 1, which, for each study, provides information on the country of implementation, study design, sample size, behaviours investigated and their measurement, network boundaries, network nomination process, SNA indicators calculated, and the overall assessed study quality. The studies were conducted in diverse settings, including secondary schools, universities, workplaces, and local communities, and involved populations of varying ages and socio-demographic characteristics. All studies collected sociometric data, which allowed the reconstruction of full or near-complete social networks within bounded populations, enabling the analysis of structural properties and relational mechanisms influencing health behaviours.

**Table 1:** Summary of the 27 studies included in the review of Whole Network Analysis applied to health behaviours.

Reference	Country	Study design	Sample size	Behaviours and measurement	Network boundary	Network nomination process	SNA indicators	Study quality
Abel et al. (65)	New Zealand	Observational - cross-sectional - mixed-methods	*n* = 267	Smoking - self-reported	Single	Directly nominated: Best friends (n = not limited)	Density, reciprocity, popularity, homophily, perceived friend behaviour	Robust
Aloise-Young and Kaeppner (64)	United States	Observational - longitudinal - quantitative	*n* = 1,630	Smoking - self-reported	Several	Roster: People whom they usually hang around with (*n* = 5) People whom they do not want to hang around with (*n* = 5)	Popular, rejected, neglected, controversial, average	Robust
Alsayed et al. (73)	Bahrain	Observational - cross-sectional - quantitative	*n* = 673	Physical activity, diet, sedentary behaviour, sleep deprivation - self-reported	Single	Directly nominated: Closest friends (*n* = 5)	Density, popularity, centrality	Robust
Arias Ramos et al. (75)	Spain	Observational - cross-sectional - quantitative	*n* = 235	Overweight, obesity - anthropometric measurements	Single	Roster: Time spends with your classmates (n = not limited)	Density, in-/out-degree, closeness, betweenness, eigenvector centrality	Robust
Arias Ramos et al. (76)	Spain	Observational - cross-sectional - quantitative	*n* = 235	Overweight, obesity - anthropometric measurements	Single	Roster: Time spends with your classmates (n = not limited)	In-/out-degree betweenness, closeness, eigenvector	Robust
Balestrieri et al. (59)	United States	Observational - cross-sectional - quantitative	*n* = 1,341	Alcohol - self-reported	Single	Roster: Students who were important to them in the past month (*n* = 10)	In−/out-degree mutuality, ties who were self-reported drinkers or perceived to be drinkers	Acceptable
Barnett et al. (56)	United States	Trials - RCT	*n* = 1,236	Alcohol - self-reported + ego’s perception	Several	Roster: Individuals in the first-year class who had been important to them in the past 30 days (*n* = 10)	Influence, selection, Jaccard Index, stochastic actor-based model indicators, homophily, tie strength, perceived peer behaviour	Strong
Barnett et al. (66)	United States	Observational - longitudinal - quantitative	*n* = 129	Substance use (alcohol, marijuana), physical activity (exercise) – self-perception	Single	Directly nominated: People who have been important to you since the start of the school year (*n* = 10) Roster: Individuals who lived in their residence hall (*n* = 10)	Community detection, network components, autocorrelation, peer influence relations, demographic and behavioural clusters, peer characteristics	Strong
Bevelander et al. (61)	Netherlands	Observational - longitudinal - quantitative	*n* = 953	Physical activity, diet, media use - self-reported + spatial analysis of social relationships	Single	Directly nominated: Friends, role models, influencers (in diet, physical activity, media use), detecting role models, impression managers, and messengers (n = not limited)	Traditional centrality measures	Robust
Bot et al. (81)	Netherlands	Observational - cross-sectional – quantitative + observational - longitudinal - quantitative	*n* = 1,280	Alcohol - self-reported	Single	Directly nominated: Best friends (*n* = 5) Most popular persons of their class (*n* = 5) Least popular persons of their class (*n* = 5)	Popularity, reciprocity	Robust
Ennett et al. (31)	United States	Observational - cross-sectional - quantitative	*n* = 1,092	Smoking - self-reported	Single	Directly nominated: Best friends (*n* = 3)	Cliques, isolates, connectedness, heterogeneity, homogeneity, homophily	Robust
Gallupe (58)	United States	Observational - longitudinal - quantitative	*n* = 13,539	Alcohol - self-reported	Single	Roster: Male friends (*n* = 5) Female friends (*n* = 5)	Popularity, centrality	Strong
Gallupe and Bouchard (57)	United States	Observational - longitudinal - quantitative	*n* = 13,351	Alcohol - self-reported	Single	Not specified. Refers to the primary Add Health research data.	Experienced benefits, density, popularity, Bonacich centrality, reach in three steps, peer alcohol use	Strong
Go et al. (67)	United States	Observational - longitudinal - quantitative	*n* = 2065	Smoking - self-reported	Single	Not specified. Refers to the primary Add Health research data.	Degree, peer selection/de-selection, direct/indirect peer influence	Robust
Hill (68)	Australia	Observational - cross-sectional - quantitative	*n* = 186	Smoking – self-perception	Single	Roster: Who do you spend most of your time with? (*n* = 5)	Homogeneity	Robust
Huisman and Bruggeman (78)	Netherlands	Observational - longitudinal - quantitative	*n* = 961	Smoking - self-reported	Several	Directly nominated: Friends (*n* = 15)	Basic rate parameter (friendship), out-degree, reciprocity, transitive triplets, Jaccard Index, gender similarity, smoking (alter, ego, similarity)	Robust
Hunter et al. (74)	Northern Ireland	Trials - quasi-experimental design	*n* = 406	Physical activity - timestamp data	Single	Sensor data collection	Density, degree centrality, triadic census, social ties, Jaccard index	Robust
Lorant and Nicaise (77)	Belgium	Observational - cross-sectional - quantitative	*n* = 487	Alcohol - Self-reported	Single	Roster: Being friends of, room-mate of, studying or working with, spending leisure time with (n = not limited)	In-degree centrality, closeness, social capital (effective size), gender-heterophily (Krackhardt Index)	Robust
Lorant and Tranmer (69)	Belgium, Finland, Germany, Italy, Netherlands, Portugal	Observational - cross-sectional - quantitative	*n* = 11,015	Smoking, alcohol, cannabis, and physical activity - self-reported	Single	Roster: Best and closest friends (*n* = 5)	Popularity, betweenness closeness	Robust
Meisel et al. (70)	United States	Observational - cross-sectional - quantitative	*n* = 972	Alcohol - self-reported	Single	Roster: Who in the first year class was important to them (*n* = 10)	In-/out-degree, global popularity, betweenness centrality, ego density, presence at maximum drinking day events, drinking behaviour and buddies, peer drinking, demographic/social factors	Robust
Perkins et al. (82)	Uganda	Observational - cross-sectional - quantitative	*n* = 719	Alcohol - self-reported	Single	Census list: Residents with whom a participant directly interacted (*n* = 6)	Personal network size, exposure to norms, norm misperception, exposure to social norm	Robust
Prinstein et al. (80)	United States	Observational - longitudinal - quantitative	*n* = 336	Smoke, aggressive behaviour - self-reported	Single	Roster: Peers who were most/least popular (*n* = not limited)	Popularity, reciprocity	Robust
Prochnow et al. (72)	United States	Observational - longitudinal - mixed-methods	*n* = 182	Physical activity - self-reported	Single	Roster: People you hang around with, talk to, and do things with the most here (*n* = 5)	Density, reciprocity, transitivity	Robust
Ramírez-Ortiz et al. (60)	Mexico	Observational - longitudinal - quantitative	*n* = 399	Smoking - self-reported	Single	Directly nominated: Best friends (*n* = 6)	In-/out-degree, In-/out-closeness, centrality, density, peer influence, homophily, social norms	Strong
Stearns et al. (79)	Canada	Observational - cross-sectional - quantitative	*n* = 706	Physical activity - self-reported + pedometer data collection	Single	Directly nominated: Close friends (*n* = 10) Best friends, (*n* = 5)	Reciprocity, degree, clustering, centrality, homophily, tie strength	Robust
Urberg et al. (62)	United States	Observational - longitudinal - quantitative	*n* = 1,028	Alcohol, smoking - self-reported	Several	Directly nominated: Best friends in school (*n* = 10) Other good friends in school (*n* = 10)	Reciprocity, clustering, group stability, dyadic-group overlap, selection, influence, substance use transition, stability effects, grade, gender, exposure	Robust
Zhang et al. (63)	United States	Observational - longitudinal - quantitative	*n* = 624	Overweight + BMI - self-reported	Single	Not specified. Refers to the primary Add Health research data.	Outdegree, reciprocity, transitive triplets, BMI sociability and attractiveness, friendship rate, BMI rate, homophily	Acceptable

Rather than organising the findings by specific behaviour, we present the results according to the five key structural mechanisms presented in the previous section.

We adopted this approach to emphasise the cross-cutting network dimensions that underlie the studies. Since our primary interest lies in the relational structures captured by WNA, this organisation better highlights the social processes through which network properties influence different behavioural outcomes.

### Peer influence and behavioural convergence

Several of the 27 studies examined peer influence as a driver of health behaviour change, particularly for alcohol use and physical activity. Influence was typically assessed through changes in individual behaviour resulting from exposure to peers within bounded settings (e.g., school classes, university cohorts).

In a randomised trial, Barnett et al. (56) applied dynamic network analysis and the Jaccard index, finding that students with more drinking peers were more likely to increase their own alcohol use, clear evidence of peer-driven convergence. Similarly, Gallupe and Bouchard (57) observed that adolescents with higher centrality and popularity were both more influential and more likely to adopt peer behaviours, reinforcing group norms.

Furthermore, Gallupe (58) also linked affiliation with higher-status peers to greater risk-taking, suggesting that influence effects are magnified by social hierarchy. While influence was not always labelled directly, studies like Balestrieri et al. (59) referenced metrics such as outdegree and peer salience, indicating similar convergence mechanisms.

Behaviours visible in shared environments—like drinking or exercising—were more subject to influence, while more private practices (e.g., diet) showed weaker peer effects.

### Selection and homophily

Selection mechanisms—forming ties with similar others—were evident in many studies, especially those with longitudinal designs. Behavioural homophily (e.g., in alcohol use, smoking, or physical activity) shaped both tie formation and maintenance, reinforcing behavioural clustering.

In their randomised controlled trial on college freshmen, Barnett et al. (56) found that students maintained friendships with peers sharing similar drinking habits, highlighting homophily as key to network stability. Similarly, Gallupe and Bouchard (57) showed that adolescents preferred peers with similar alcohol use, especially within central and popular positions.

Ramírez-Ortiz (60), examining adolescent smoking, found that youths formed behaviourally homogenous subgroups based on smoking status and demographic similarity. Likewise, Bevelander et al. (61) reported that adolescents’ physical activity levels predicted friendship choices, reinforcing health-related segmentation.

Even in behaviour-change interventions involving peer models, selection patterns persisted. Although not all studies modelled selection explicitly, the consistent clustering of behaviours across networks supports its widespread influence.

### Network cohesion and subgroup effects

Several studies highlighted how cohesive network structures, including high density, transitivity, and clustering, can reinforce behavioural norms within peer groups. Cohesion was often associated with behavioural stability, as individuals embedded in tightly knit networks tended to conform to prevailing group practices.

For example, Gallupe and Bouchard (57) found that adolescents in dense friendship networks were more likely to adopt group-aligned drinking behaviours, indicating a normative reinforcement effect. Similarly, Balestrieri et al. (59) reported that clustering around peer leaders influenced the spread of physical activity behaviours in university settings.

Some studies also pointed to segmented subgroups within larger networks, especially when cohesion intersected with homophily (31, 60, 62, 63). This produced behaviourally distinct clusters that maintained internal consistency but differed from one another, limiting behaviour diffusion across the broader network.

The dual role of cohesion emerged clearly: while it can promote healthy habits within aligned groups, it may also entrench risk behaviours when those are dominant. These effects underscore the importance of structural integration and normative orientation in shaping behaviour trajectories.

### Centrality and behavioural diffusion

Network centrality—measured through indicators like in-degree, closeness, and betweenness—was a strong predictor of behavioural diffusion across multiple studies. Highly central individuals were more likely to influence peers and set behavioural norms.

In school settings, Gallupe and Bouchard (57) observed that popular adolescents with high centrality scores played a key role in spreading alcohol use. Likewise, Balestrieri et al. (59) noted that students connected to visible peer leaders were more likely to adopt shared activity behaviours.

Central actors also impacted intervention outcomes. Their normative alignment—whether promoting or discouraging risky behaviours—shaped their effectiveness. In some cases, high-centrality individuals served to stabilise behaviours rather than drive change, especially when their attitudes were conservative or resistant to innovation.

Overall, centrality functions both as a conduit and a gatekeeper in the behavioural diffusion process, depending on context and the perceived legitimacy of central actors.

### Brokerage and bridging roles

Although less frequently analysed, brokerage, the role of individuals who link otherwise disconnected subgroups, emerged as a relevant but underexplored mechanism in several studies.

Balestrieri et al. (59) noted that certain students acted as connectors between peer groups, influencing how physical activity behaviours spread across university networks. These bridging roles enabled the diffusion of behaviours beyond tightly clustered subgroups, supporting broader reach.

However, brokers’ effectiveness often depended on their social capital and credibility in each group. In contexts where norms differed between clusters, brokers could experience tension or reduced influence, limiting their potential as change agents.

Few studies in the review formally measured brokerage using structural metrics (e.g., betweenness centrality or constraint), indicating limited empirical attention to brokerage processes within the current literature.

### Summary of key patterns

Across the 27 studies, five structural mechanisms consistently shaped health behaviours:

- Peer influence promoted behavioural convergence, particularly in visible or shared contexts like schools or dormitories.
- Selection and homophily led individuals to form and maintain ties with behaviourally similar peers, reinforcing clustering.
- Network cohesion amplified group norms, either healthy or risky, depending on the subgroup’s dominant behaviour.
- Centrality was associated with increased influence; central actors often drove diffusion or reinforced norms.
- Brokerage facilitated cross-group transmission but remained underexplored and context-dependent.

These findings highlight the added value of WNA in uncovering the social architecture underlying health behaviours, insights often missed by individual-level approaches.

## Discussion

This systematic review synthesised empirical studies that applied WNA to investigate how social structures influence key health behaviours: smoking, alcohol use, physical activity, and diet. Our aim was to evaluate the unique contribution of WNA to understanding behavioural diffusion and clustering, identify the relational mechanisms most consistently observed, and outline the methodological and substantive value of adopting a whole-network perspective in public health research.

The results confirmed that WNA enables the detection of a set of recurrent structural mechanisms (peer influence, selection and homophily, subgroup cohesion, centrality, and brokerage) that shape the emergence and maintenance of health behaviours within defined populations. Unlike traditional individual-level approaches or even egocentric network models, WNA allows for a more complete understanding of the relational embeddedness of health practices, uncovering how behaviours are not only adopted and sustained, but co-produced within social fields.

### Whole network analysis as a distinctive lens

One of the main findings of this review is the unique capacity of WNA to model behavioural processes at the level of entire social systems. While egocentric approaches capture perceived influences and support, WNA identifies actual structural patterns such as clustering, transitivity, and centrality that condition both exposure to behaviours and susceptibility to change. By reconstructing full networks, WNA enables researchers to map how behaviours circulate through specific pathways and to locate where in the network behavioural reinforcement is strongest.

For example, in several reviewed studies, students’ health behaviours were more strongly predicted by their position in the network, such as centrality or popularity, than by individual traits or demographic factors. Gallupe and Bouchard (57), studying alcohol use among adolescents, found that students with higher sociometric status were more susceptible to adopting dominant behaviours within their networks, indicating that embeddedness in peer hierarchies significantly shaped behavioural outcomes. Similar evidence on sociometric status and smoking onset was observed in Aloise-Young and Kaeppner (64), who used roster nominations and sociometric categories (popular, rejected, neglected) to show that status predicted progression in cigarette smoking. Abel et al. (65) further illustrated, using best-friend nominations and metrics such as density, reciprocity and homophily, how friendship structures relate to smoking behaviour—highlighting that even in cross-sectional classroom samples, tie patterns and perceived friend behaviour are strongly associated with smoking prevalence.

Moreover, WNA makes it possible to explore both peer influence and selection mechanisms, particularly in studies with longitudinal designs. Some studies, such as Barnett et al. (56), suggest that social ties and health behaviours can co-evolve over time, highlighting the dynamic relationship between individual behaviour and network structure. Earlier longitudinal work by Barnett et al. (66) similarly used community detection, autocorrelation and peer-association metrics to show concurrent clustering of substance use and exercise behaviour within college social networks, providing evidence that affiliation patterns and behavioural clusters can emerge and persist across time.

Consistently, Go et al. (67) used longitudinal Add Health data and degree-based measures to document patterns of peer selection and social distance in smoking initiation, illustrating how homophily and tie formation jointly shape smoking trajectories. Hill’s (68) early roster-based work also provided foundational evidence that affiliation patterns within small groups relate to adolescent smoking conformity.

### The relational logic of health behaviour transmission

A central contribution of the studies reviewed is the demonstration that health behaviours follow a relational logic; that is, behaviours are not simply correlated among individuals but are shaped by structural conditions, as discussed in the following subsections:

#### Clustering

Behaviours tend to cluster within cohesive subgroups, where local norms are reinforced, and deviance from group expectations is discouraged (56, 57, 62, 69, 70). High clustering was associated with both risk (e.g., smoking groups) and protective effects (e.g., sports team-based physical activity). As demonstrated in prior studies, behaviours tend to spread through social networks beyond direct interpersonal relationships (10, 11). Ennett et al. (31) contributed early empirical evidence on variability of smoking within and between adolescent cliques, using clique and connectedness metrics to highlight heterogeneity in smoking prevalence across dense subgroups. Similarly, Ramírez-Ortiz et al. (60) applied traditional centrality and density measures in Mexican high-school networks to show peer influence and homophily for tobacco use, evidencing clustering of smokers within friendship cliques.

#### Centrality

Individuals with higher in-degree or betweenness centrality had greater visibility and were more likely to model behaviours for others. These actors also played a key role in amplifying interventions, especially when targeted as “peer leaders” (71). Studies found that network density, degree centrality, and clustering significantly influence how behaviours spread within social groups (72–74). Arias et al. (75, 76) used degree, closeness and eigenvector measures in classroom rosters to show that socio-metric centrality correlated with overweight/obesity and social exclusion patterns, indicating how central actors can both reflect and perpetuate health-related social stratification. Gallupe (58), focusing on social status and alcohol, further emphasized how positional benefits (centrality/popularity) relate to motivation for alcohol use. Lorant and Nicaise (77) added to this evidence by employing in-degree, closeness and effective-size (social capital) measures in a university binge-drinking study to demonstrate that students’ centrality and network effective size related to binge episodes and that gender-heterophily modified those associations—showing how centrality interacts with composition to shape drinking risk.

#### Brokerage

Although brokerage is conceptually relevant for understanding the diffusion of cross-group behaviour, few studies in our sample explicitly analysed brokerage roles or computed related metrics. This points to a methodological gap and suggests the need for future research to examine how individuals who span structural holes may facilitate or hinder the spread of health behaviours across network segments. The limited use of brokerage measures may also reflect practical and methodological challenges. Brokerage roles are often difficult to identify reliably in incomplete networks because missing relational data can substantially alter estimates of bridging positions. In addition, brokerage effects may vary across contexts depending on subgroup boundaries, institutional structures, and the stability of social ties over time. These challenges likely contribute to the relative underuse of brokerage-focused analyses despite their theoretical relevance for understanding behavioural diffusion across otherwise disconnected social groups. Notwithstanding the scarcity of explicit brokerage metrics, Huisman and Bruggeman (78) used transitive triplets and reciprocity parameters in longitudinal models to indirectly highlight how structural configurations (including non-closed ties) relate to smoking dynamics, while Zhang et al. (63) used agent-based models parameterized on Add Health measures (outdegree, reciprocity, transitive triplets) to simulate how cross-group ties could influence adolescent BMI dynamics, suggesting brokerage-like pathways in weight-related diffusion.

These relational mechanisms cut across behaviour types, suggesting that smoking, diet, alcohol use, and physical activity are all susceptible to similar structural dynamics. While the visibility and immediacy of certain behaviours (e.g., smoking or exercising together) may modulate the strength of influence, the underlying network patterns, such as clustering and selection, remain consistent. Balestrieri et al. (59) compared drinkers and nondrinkers on network characteristics (in/out-degree, mutuality, perceived drinking ties) showing that perceived exclusion and network position jointly related to drinking status among college students. Bevelander et al. (61), in the MyMovez protocol, illustrated how role models and impression managers—identified through open nominations and centrality measures—matter for diet and physical activity norms among youth. Stearns et al. (79), combining pedometer data with friendship nominations, provided objective corroboration that ties and clustering in children’s networks correspond with both in-school and out-of-school physical activity levels. Prinstein et al. (80) demonstrated longitudinal associations between popularity (measured through peer nominations) and risk behaviours (including smoking and aggression), underscoring non-linear status-behaviour relationships. Bot et al. (81) and Urberg et al. (62) each documented how specific friendship selection and popularity nominations (best friends, most/least popular) moderate adolescent alcohol use, adding to the cross-study consistency on nomination framing and observed behavioural clustering. Perkins et al. (82), working in a whole-population rural Ugandan sample and using census-style interaction nominations, showed how norm misperception and exposure metrics relate to drinking behaviours at the village level, demonstrating WNA utility beyond school settings. Finally, Prochnow et al. (72) and Meisel et al. (70) together illustrate how mixed methods and event-level analyses can enrich our understanding of when and where clustering and peak events (e.g., maximum drinking days) occur, using in/out-degree, ego-density and event-attendance indicators.

### Advantages of WNA for public health research and practice

The synthesis highlights several advantages of WNA for both theoretical development and public health practice:

- Improved explanation and prediction: WNA can account for variance in behaviours that traditional models miss, especially when peer structures play a stronger role than individual traits. This allows for better predictive models and more realistic causal assumptions.
- Detection of hidden subpopulations: WNA identifies subgroups that may not be captured by demographic segmentation but are crucial for behavioural intervention (e.g., informal cliques, risk clusters).
- Design of targeted interventions: WNA enables the strategic targeting of interventions to highly central individuals or influential subgroups, maximising the diffusion potential of messages or norms.
- Monitoring of intervention spread: In longitudinal designs, WNA can track how behaviours or health messages spread through a network, allowing for dynamic assessment and adjustment.
- Cross-level integration: WNA can be combined with individual-level variables and contextual factors, supporting multilevel modelling of behaviour.

Given these strengths, WNA represents a methodological innovation that is underutilised relative to its potential. The review suggests that expanding the use of WNA could enhance both theory-building in social epidemiology and practice-oriented designs in health promotion.

### Limitations in current applications of WNA

Despite its potential, several limitations and gaps were noted across the studies reviewed:

- Overreliance on cross-sectional designs: Many studies used cross-sectional data, which limits the ability to distinguish between influence and selection. Longitudinal designs (e.g., panel data, SAOMs) are better suited to disentangling these processes.
- Underuse of brokerage metrics: Although centrality and density were widely used, metrics related to brokerage and structural holes were rarely applied. This reflects a missed opportunity to understand how network bridges facilitate or hinder behaviour diffusion.
- Limited attention to contextual moderators: Few studies examined how broader contextual factors, such as school culture, policy environments, or socioeconomic inequalities, interact with network processes. This restricts the ecological validity of network findings.
- Lack of replication and comparability: Variability in network definitions (e.g., name generators), sample sizes, and metrics used complicate cross-study comparisons. Establishing methodological standards for WNA in health behaviour research would enhance cumulative knowledge.
- Narrow behavioural focus: Most studies examined only one behaviour in isolation, with limited exploration of behavioural co-occurrence or substitution (e.g., sedentary behaviour versus diet). Future research could benefit from modelling behavioural multiplexity.

### Future directions

Based on the findings of this review, we recommend several directions for advancing the use of WNA in health behaviour research:

- Expand WNA to diverse settings and populations: Most studies focused on adolescents in school contexts. Research should explore the application of WNA in everyday life settings, such as workplaces, local communities, and towns, where relational mechanisms may operate differently and offer unique insights into health behaviour dynamics.
- Integrate WNA with social determinants of health: Network structures are shaped by and contribute to social inequality. Integrating WNA with analyses of SES, race, and gender can uncover how health disparities are reproduced relationally.
- Use WNA in intervention design and evaluation: Public health interventions should routinely assess network structure before and after implementation. Pilot programs can test whether network-informed designs are more effective in promoting behaviour change.
- Develop hybrid models: Combining WNA with qualitative methods, agent-based modelling, or ecological systems theory can enrich understanding of multilevel health influences.
- Promote open data and replicability: Publishing anonymised network data and analytic code would support transparency, cross-study comparison, and methodological refinement.

### Contributions of the review

This review contributes to the literature by providing the first systematic synthesis of WNA applied to multiple health behaviours in peer-reviewed empirical research. Unlike previous reviews that focus on egocentric networks or specific behaviours (e.g., smoking only), this synthesis offers a comparative, cross-behavioural perspective on the structural mechanisms underlying health behaviour transmission.

It clarifies the added value of whole-network designs, both in terms of explanatory power and practical relevance, and points to specific mechanisms that are consistently implicated across behavioural domains.

Moreover, the review identifies gaps in the field, including methodological underuse of brokerage, limited contextualization, and the need for standardisation. By addressing these issues, future research can build a more cumulative, socially grounded understanding of health behaviour dynamics.

### Strengths and limitations

A major strength of this review lies in its systematic and comparative approach, focusing on multiple health behaviours across diverse populations. By applying rigorous PRISMA criteria and emphasizing structural mechanisms, the review offers a coherent synthesis of how WNA contributes to public health knowledge. The emphasis on relational processes rather than isolated behaviours allows for a more integrative understanding of social influences on health.

Another strength is the review’s focus on whole-network designs, which remain relatively rare but offer powerful explanatory leverage. By excluding studies that rely solely on egocentric data or unstructured relational measures, the review maintains a high standard of methodological consistency.

However, the review has limitations. First, the exclusion of non-English articles and grey literature may have led to the omission of relevant studies. Second, the heterogeneity in metrics, models, and definitions across studies made meta-analytic synthesis unfeasible. Third, while the review identifies structural mechanisms, it does not evaluate the effectiveness of specific interventions, which would require a different methodological framework.

Finally, although the review identifies key mechanisms, it does not fully address how social inequalities interact with network processes. Future work should deepen this dimension, integrating structural determinants with relational analyses.

## Conclusion

This systematic review underscores the critical importance of WNA in understanding the social transmission of health behaviours such as smoking, alcohol use, physical activity, and dietary patterns. Unlike individual-centric models or egocentric network designs, WNA captures the relational architecture of behaviours within fully mapped social systems, revealing how behaviours cluster, spread, and stabilize through patterned interactions.

The reviewed studies collectively demonstrate that health behaviours are deeply embedded in social networks and influenced by structural dynamics such as peer influence, homophily, cohesion, and centrality. These findings suggest that interventions and policies aiming to promote healthy behaviours must move beyond individual-level determinants and address the social mechanisms and structures that support or constrain change.

Moreover, WNA enables the identification of influential actors and cohesive subgroups, offering strategic insights for designing targeted and scalable interventions. Longitudinal applications of WNA, particularly those using advanced modeling techniques, offer the most powerful tools for understanding the co-evolution of networks and behaviours.

Despite its promise, WNA remains underutilized in public health research. This review calls for broader application of WNA across diverse settings and populations, greater integration with social determinants of health, and increased methodological transparency. The relational insights offered by WNA are indispensable for crafting interventions that are socially attuned, structurally grounded, and more likely to achieve sustained behavioural change.

In conclusion, WNA represents a paradigm shift in the study of health behaviours. By placing individuals within their full relational contexts, this approach enables researchers and practitioners to understand how health is not merely acted upon by individuals but emerges from social systems. Embracing this perspective is essential for advancing both scientific understanding and public health practice in an increasingly interconnected world.

## Data Availability

The original contributions presented in the study are included in the article/supplementary material, further inquiries can be directed to the corresponding author.
